# A Multiscale Overview of Modelling Rolling Cyclic Fatigue in Bearing Elements

**DOI:** 10.3390/ma15175885

**Published:** 2022-08-26

**Authors:** Muhammad U. Abdullah, Zulfiqar A. Khan

**Affiliations:** 1 Department of Mechanical Engineering, University of Bristol, University Walk, Bristol BS8 1TR, UK; 2 NanoCorr, Energy & Modelling (NCEM) Research Group, Department of Design & Engineering, Bournemouth University, Poole BH12 5BB, UK

**Keywords:** rolling fatigue, bearing steel, cyclic hardening, microstructure, residual stresses

## Abstract

During service, bearing components experience rolling cyclic fatigue (RCF), resulting in subsurface plasticity and decay of the parent microstructure. The accumulation of micro strains spans billions of rolling cycles, resulting in the continuous evolution of the bearing steel microstructure. The bearing steel composition, non-metallic inclusions, continuously evolving residual stresses, and substantial work hardening, followed by subsurface softening, create further complications in modelling bearing steel at different length scales. The current study presents a multiscale overview of modelling RCF in terms of plastic deformation and the corresponding microstructural alterations. This article investigates previous models to predict microstructural alterations and material hardening approaches widely adopted to mimic the cyclic hardening response of the evolved bearing steel microstructure. This review presents state-of-the-art, relevant reviews in terms of this subject and provides a robust academic critique to enhance the understanding of the elastoplastic response of bearing steel under non-proportional loadings, damage evolution, and the formation mechanics of microstructural alterations, leading to the increased fatigue life of bearing components. It is suggested that a multidisciplinary approach at various length scales is required to fully understand the micromechanical and metallurgical response of bearing steels widely used in industry. This review will make significant contributions to novel design methodologies and improved product design specifications to deliver the durability and reliability of bearing elements.

## 1. Background

Rolling bearing elements (RBEs) are fundamental parts of the automotive industry, turbines, and precision machinery. Bearings are an integral part of different mechanisms and assemblies. RBEs are used to transfer loads and motion while providing structural support to the machine components [1]. A bearing element consists of an outer part and an inner part which are connected to the machine parts and several rolling elements. Bearing elements are categorised depending upon the roller type (i.e., cylindrical roller, spherical roller, tapered roller) and can be used based on load and lubrication requirements. During service, rolling bearing elements are subjected to highly varying loads in high-speed turbines, precision machinery, automotive, and aerospace industries. The rolling bearing elements are operated under non-proportional loadings over hundreds of millions of stress cycles. The bearing material response under RCF is dependent on many factors, i.e., contact stress, frictional coefficient, residual stresses, carbide volume fraction, inclusions, and bearing material microstructure [2]. During operation, microstructure, microtexture, compressive residual stresses, and localised hardness in the subsurface area evolve with progressing stress cycles [3,4], making the RCF phenomenon much more complex and heterogeneous. Moreover, non-metallic inclusions, surface defects, slippage, and starved lubrication conditions create further scatter and uncertainty in the service life of a rolling element [5,6].

Rolling contact fatigue (RCF) in bearings is manifested as subsurface plastic damage accumulated under the rolling contact path. This subsurface damage is highly localised and is accumulated at the microstructural scale [7] (typically in the vicinity of carbides due to stress concentration). The ability of a material to strain-harden with the continuous accumulation of plasticity is very high where the material yield point translates (kinematic hardening) and expands (isotropic hardening). Therefore, the continuous evaluation of material hardening/softening becomes critical for researchers to acquire the cyclic hardening response of evolved bearing steel, which is quite difficult to predict with simplistic material hardening models. It is well known that this subsurface plastic deformation involves a forward flow of material parallel to rolling direction, which causes compressive residual stresses in the axial and circumferential direction [8,9]. The deep zone residual stresses arise due to the decomposition of parent microstructure and accumulation of non-uniform plasticity [10] during RCF. Earlier studies [3,11,12] reported that the life span of bearing elements during operation can be altered due to deep zone compressive residual stresses (CRS). The CRS help in the closure of crack propagation by impeding the stress concentration at the notch [12,13]. It can also affect the amplitude of applied stress amplitude. During microplasticity, the microstructure of the subsurface region evolves, and the material phase transformation takes place, resulting in formation of microstructural features, i.e., dark etching regions (DERs) and white etching bands (WEBs). Dark etching regions (DERs) are observed as black patches under an optical microscope after etching with Nital (3% nitric acid solution in ethanol) solution. DERs are believed to be a tempered form of martensite [14] and are formed much earlier than white etching bands (WEBs). WEBs, in contrast to DERs, are developed in the later life of RCF and appeared as bright contrast under an optical microscope. The WEBs are considered ferritic in nature and remain throughout the RCF cycles. These elongated white bands are reported as rectangular disc-shaped, which are inclined at an angle of approximately 30° and 80° with the contact path and are termed as lower angle bands (LABs) and higher angle bands (HABs), respectively [15,16,17]. The scanning electron microscopy (SEM)—along with electron backscattering diffraction (EBSD)—investigations revealed that WEBs are shear bands which are formed at a depth of principal and orthogonal shear maxima [18,19]. The redistribution of the carbon atoms during RCF was reported as the significant factor to drive this phase transformation phenomenon. Despite the advancements in the material characterisation technique, resulting in new aspects of the localised RCF damage, researchers are still unable to comprehend the formation mechanism of such microstructural features [20,21]. The stress component responsible for DERs formation, along with the unique directionalities of WEBs, have been up for debate. Moreover, it has been reported [22,23] that close proximity exists between CRS and depth distribution of microstructural features (i.e., DERs, WEBs); however, how the microstructural alterations are affected by the presence of compressive stresses has not been quantified.

## 2. Bearing Steel

**Figure 1:**
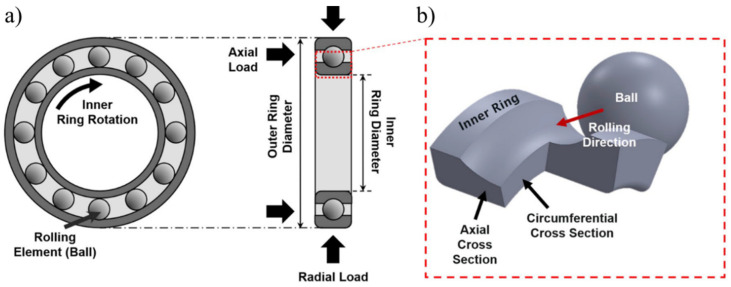
(**a**) Schematic of a deep groove ball bearing subjected to axial and radial load; (**b**) axial and circumferential cross-section of the inner ring is demonstrated [2].

Rolling contact fatigue (RCF) is quite distinct from conventional uniaxial fatigue (i.e., low and high cyclic fatigue), where a bulk of specimen material is tested to generate the stress ‘S’ versus cycles to failure ‘N’ (known as S–N curves) [20]. These two regimes of fatigue are defined by the number of cycles to failure. The low cycle fatigue (LCF) usually operates at higher loads, where the plasticity is accumulated homogenously, appears widespread, and is characterised by repeated plasticity (plastic deformation in each cycle). The high cycle fatigue (HCF) is characterised by elastic deformations, where the stress levels are below the macroscopic yielding, and plasticity is accumulated in the isolated regions under loading (i.e., near non-metallic inclusion due to stress concentrations). There is no fixed transition line where the two regimes can be separated, as this transition depends upon material ductility [24]. RCF, in contrast, occurs as a highly localized plastic damage in bearing elements, where the rated life is typically expressed in billions of rolling cycles. This plastic damage is accumulated under the rolling contact path and can be investigated in the axial and circumferential section of bearing components, as illustrated in Figure 1. 

**Figure 2:**
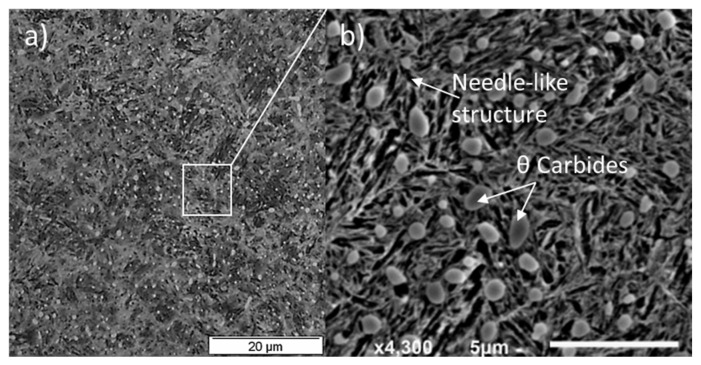
(**a**) Microstructure of martensitic bearing steel; (**b**) the needle-like structure containing primary θ carbides, adapted from [29].

The standard process to manufacture martensitic steel is the partial austenitization of steel, which consists of austenite and almost 4 vol% cementite (θ carbides). The austenite phase is followed by the quenching process, where almost 10–11% of the austenite phase remains in the martensitic matrix due to high carbon content and is termed as retained austenite (RA) [25]. During rapid cooling, the carbon cannot diffuse out of the solid solution and forms a supersaturated martensitic matrix in a body-centred tetragonal (BCT) structure. It is reported [26] that the quenched martensitic solid solution contains internal stress due to material phase transformations, along with resulting volume expansion/contraction, and is compensated by a large number of dislocations within the matrix. The resulting martensitic structure can attain hardness as high as 900 HV, but is highly brittle and contains internal residual stresses. The brittleness of the microstructure can be overcome by tempering the steel at 200 °C for 1–2 h to reduce the hardness and introduce ductility. During tempering, carbon leaves the solid solution and creates nanosized precipitates (ε and η), also called tempered carbides, or transition carbides. The size and morphology of these transition carbides depend upon the tempering condition. The proper balance of the primary carbides (θ) and transition carbides (ε and η) in the microstructure ensures the bearing material toughness and strength [27]. Transmission electron microscopy (TEM) of the through-hardened bearing steel has revealed that the θ carbides have (Fe, Cr)3C structure and are made up of 12 wt.% chromium [28]. The addition of chromium (Cr) to the bearing steel increases the wear resistance, strength, and surface hardness. The overall microstructure of standard martensitic steel contains needle-like martensite plates, 10–11% RA, along with undissolved carbides (θ, ε, and η), which can be observed in Figure 2.

**Figure 3:**
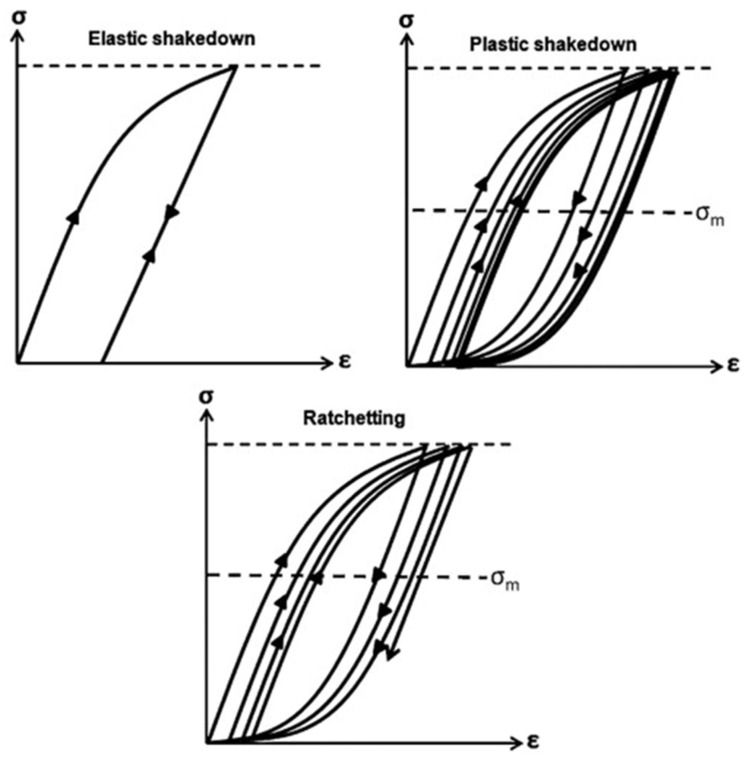
Schematic of the cyclic hardening response of bearing steel subjected to RCF [35].

The nominal stress of a bearing element during service is usually below 2.5 GPa; consequently, the applied stress is unable to cause material flow. However, at a microstructural scale, the stress amplifies due to stress intensity factor around carbides and non-metallic inclusions causing an irreversible plastic flow in the subsurface region of bearing material, which is accumulated with each rolling cycle [30,31]. It is reported [32] that the RCF-induced cyclic hardening exponent is different from the monotonic strain hardening exponent, which greatly affects the fatigue life of rolling elements. During RCF cyclic loadings, there are four types of material plasticity zones: (i) the elastic region, (ii) the elastic shakedown, (iii) the plastic shakedown, and (iv) ratcheting, as demonstrated in Figure 3. Initially, when the material is loaded elastically, it recovers back to its initial stage and is described in the elastic region. When the load exceeds the yield limit of the material, the material experiences plasticity and resulting residual stress, which resides in and is added to the subsequent loading cycles. The generation of residual stresses is manifested due to two reasons: cyclic plasticity and the transformation of RA in the parent martensitic matrix [33]. If the following cycles do not create any plasticity due to yield point offset, then the material is said to be in the elastic shakedown region. However, if the subsequent loading cycles create combined stress well above the elastic shakedown limit, then the material can experience two possible plasticity modes, e.g., plastic shakedown and ratchetting. In plastic shakedown, the material undergoes closed-loop stress–strain hysteresis, whereas in ratcheting, the material undergoes continuous accumulation of plasticity in an open hysteresis loop [34]. Generally, the RCF life is categorised into three stages: stage I as elastic/plastic shakedown, stage II, where the bearing undergoes steady-state response (which spans to billions of RCF cycles), and stage III as instability or the failure stage. The microstructural alterations, as discussed previously, are dominant in stage II of RCF.

Bearing material under cyclic loadings manifests considerable strain hardening of the subsurface region due to plasticity, which continues for billions of cycles until failure [36]. The prolonged accumulation of subsurface plasticity results in the considerable hardening of bearing material [37,38] and a change in subsurface microstructure [3,39]. During the early shakedown stage of RCF (stage I), the continuously evolving cyclic plasticity results in the build-up of residual stress, while the material yield strength increases due to significant work hardening, as explained earlier. It is noted [40] that a higher load applied at the initial stage results in a significant work hardening, which causes an extended fatigue life of bearing elements. Experimental findings [41] from subsurface hardness measurements after progressive RCF cycles have revealed that the subsurface hardening of bearing elements can persist for a longer period, up to 240 million cycles. 

Besides considerable work hardening, subsequent softening is also reported [35,42,43,44] in the later stage of RCF loadings (i.e., stage II). As mentioned previously, bearing elements under cyclic loadings experience initial plastic shakedown in the early stage of the RCF loading cycles. The shakedown limit of an elastic–plastic material is dependent on the shear cyclic yield strength ‘k’ of bearing material [8,25]. Generally, the shakedown is described in the form of $P_{\max}/k$, where $P_{\max}$ is the maximum Hertzian contact pressure. For frictionless line contact and point contact, yielding will occur if this ratio becomes equal to 4.0 and 4.7, respectively [45]. The shear cyclic yield strength ‘k’ can be estimated as k=σy3 where $\sigma_{y}$ is the tensile cyclic yield strength [45,46]. The cyclic strains which are developed after the shakedown stage are dependent on cyclic yield strength and are typically found in the range of $0\leq \varepsilon_{p}\leq 0.002$.

**Figure 4:**
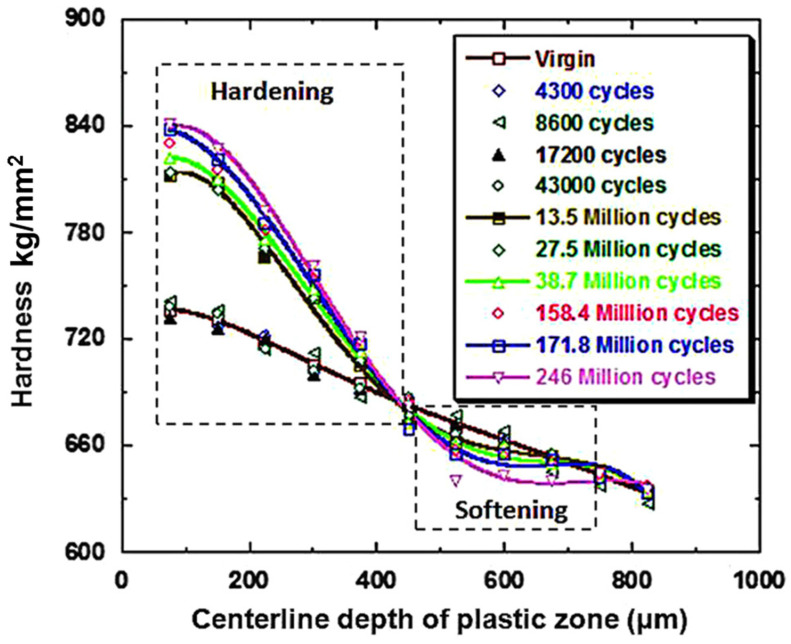
The centreline hardness is shown as a function of depth. Hardening takes place at a depth of nearly 450 µm. A softened region can also be observed at 500–700 µm depth. Maximum hardening can be observed after 246 million RCF cycles [35].

The inevitable development of microstructural alterations under contact track (driven by the diffusion of carbon in the parent structure [47,48]) and the local temperature peaks during cyclic loadings can trigger potential slip systems, resulting in material softening [20,36,49]. Moreover, for bearing elements under elastic shakedown conditions, the overall material response under loading is generally considered elastic. However, under plastic shakedown conditions, the stress–strain response of the bearing material forms a closed loop, as shown in Figure 3, where the local plastic deformations accumulate near carbides and non-metallic inclusions, causing microstructural alterations and cyclic softening, which results in further increase in the cyclic strain amplitude, leading to failure or instability (stage III) [2]. The bearing material hardening and softening response when subjected to RCF is demonstrated in Figure 4.

## 3. Material Modelling

**Figure 5:**
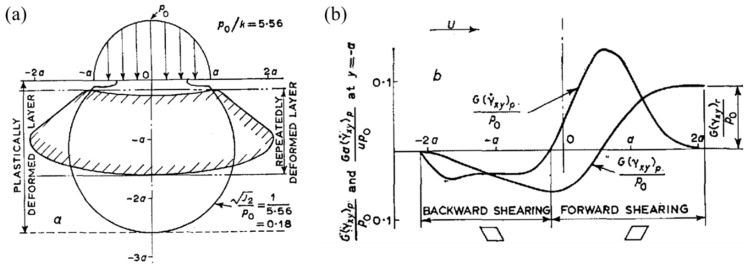
(**a**) Zone of plastic action in the steady state. (**b**) The steady cycle of plastic shear strain, indicating forwarding and backward shearing [8].

When two rollers roll together with a sufficiently high load, the subsurface region experiences plasticity, which is accumulated under the contact track and the surface layer of the cylinder, displacing in the forward direction of motion [8]. This phenomenon was initially described in 1963 [50], when it was stated that the forward flow of the surface layer of a cylinder is a result of a complex stress state, as described in Figure 5a,b. During the rolling process, compressive residual stresses are evolved in the initial first few loading cycles, provided that the load exceeds the yield limit of the material. Bhargava et al. [51] were among the early researchers to present a numerical model for RCF using a 2D finite element analysis of elastic–plastic behaviour.

The initial theoretical and numerical analyses [8,50,52,53] highlighted the linear elastic–plastic behaviour of bearing material. Later on, Howell and Bower et al. [54,55] described the isotropic and nonlinear kinematic hardening of bearing material that denotes the linear expansion and translation of the yield surface in the stress space. A three-dimensional semi-analytical code was presented by Jacq et al. [56], which was later extended by Chaise and Chen [57,58] in order to estimate the residual stress/strain after a few rolling cycles. They reported that the maximum contact stress and the equivalent plastic strains (PEEQ) were dependent on the ellipticity ratio of the interacting surfaces. The change in composition and heat treatment processes employed in the widely used bearing steels was also characterised. From cyclic push-pull experiments on the three variants of bearing steel, it was reported [46] that a standard martensitic hardened bearing steel represents pronounced hardening when subjected to cyclic loadings, when compared with the bainitic variant of steel. This is due to the fact that RA decays at a much lower stress level, leading to the onset of plastic deformation and consequently, cyclic hardening.

**Figure 6:**
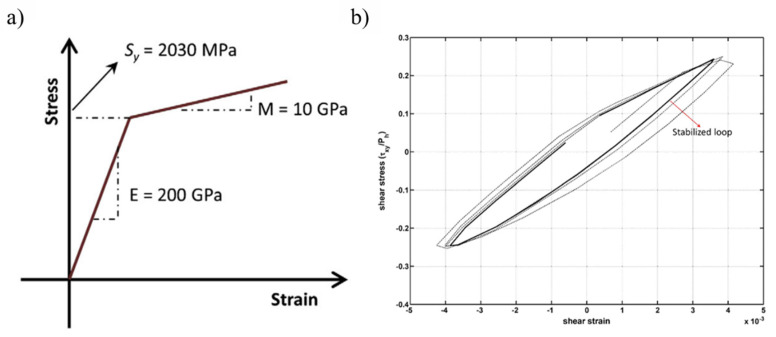
(**a**) The elastic linear kinematic plastic (ELKP) stress–strain curve acquired from the torsional testing of bearing steel; (**b**) the shear cyclic stress–strain hysteresis loop generated by multiple rolling cycles [62].

A bilinear elastic-linear kinematic plastic (ELKP) model was presented [45,59] with the help of multivalued cyclic stress–strain relationships. The material parameters utilised in the ELKP material model are elastic modulus ‘E’, yield strength ${‘S}_{y}’,$ kinematic yield strength ${‘\sigma }_{k}’,$ and plastic modulus ‘M’, which are nominally derived from extensive experimental data of fatigue tests. It is believed that the idealisation of the ELKP material model from torsional testing data is feasible for finite element analysis of rolling contact fatigue and is utilised by different researchers [10,59,60,61,62] for RCF modelling. Figure 6a shows a generic representation of the stress–strain curve for ELKP relationships whose simulation parameters are acquired from the torsional testing of standard AISI 52100 bearing steel. The resulting shear cyclic stress–strain relationship depicts a close hysteresis loop because of rolling contact loading and is represented in Figure 6b. The stabilised shear stress–strain loop (indicated in dark colour) shows the steady state response indicative of plastic shakedown. Hahn et al. [43] presented a 2D finite element model of the ELKP response, and the results were compared with the available experimental data on deep groove ball bearings (DGBB) [3,14]. The finite element simulation results, based on the torsional fatigue testing of standard bearing steel, revealed that the cyclic plasticity and modest residual stresses corroborate well with the early life of RCF (i.e., less than 10^6^ cycles). However, for the later life of RCF (i.e., 10^8^ ≤ N ≤ 10^10^ cycles), the simulation results underpredicted the formation of residual stress, in which the bearing material experienced significant metallurgical changes under RCF [43]. It was stated that the later formation of residual stresses, accompanied by metallurgical change, depends upon the relative volume fraction of the decayed microstructure. However, no direct relationship of compressive residual stresses with the subsurface microstructural alterations was considered in detail. The underprediction of calculated residual stresses from finite element simulation leads to the argument that a more complex material model is required to compensate for the mechanical-metallurgical response associated with the RCF. Later, it was also reported [63] that the bearing material subjected to rolling cycles exhibits nonlinear kinematic hardening, which can be described by the Ramberg–Osgood model. A later study [10] presented J2 plasticity-based finite element simulations incorporating linear kinematic hardening and nonlinear kinematic hardening, consecutively. Nevertheless, the cyclic behaviour of a hardened bearing under RCF is complex and challenging to define with simplistic isotropic hardening [64] and kinematic hardening [65,66,67].

To incorporate the complex hardening of bearing steel, a combined nonlinear isotropic and kinematic hardening (NIKH) material model was employed to model subsurface plasticity during cyclic loadings [68]. The NIKH material model was originally presented by Chaboche [69] and is capable of simulating various material responses under multiaxial cyclic loading, i.e., the Bauschinger effect, with mean stress relaxation, plastic shakedown, and ratcheting. Due to its complex nature, the NIKH model has been widely implemented in fatigue applications [70,71,72]. It was reported [68,73] that the NIHK material model can accurately mimic the cyclic hardening and continuous accumulation of plasticity (ratcheting) in the vicinity of carbides. However, the estimation of material constants for such a complex model is very crucial and requires significant experimental data for calibration. As discussed earlier, such material data can be acquired from conventional cyclic stress–strain curve or torsional fatigue tests of bearing steel [74].

**Figure 7:**
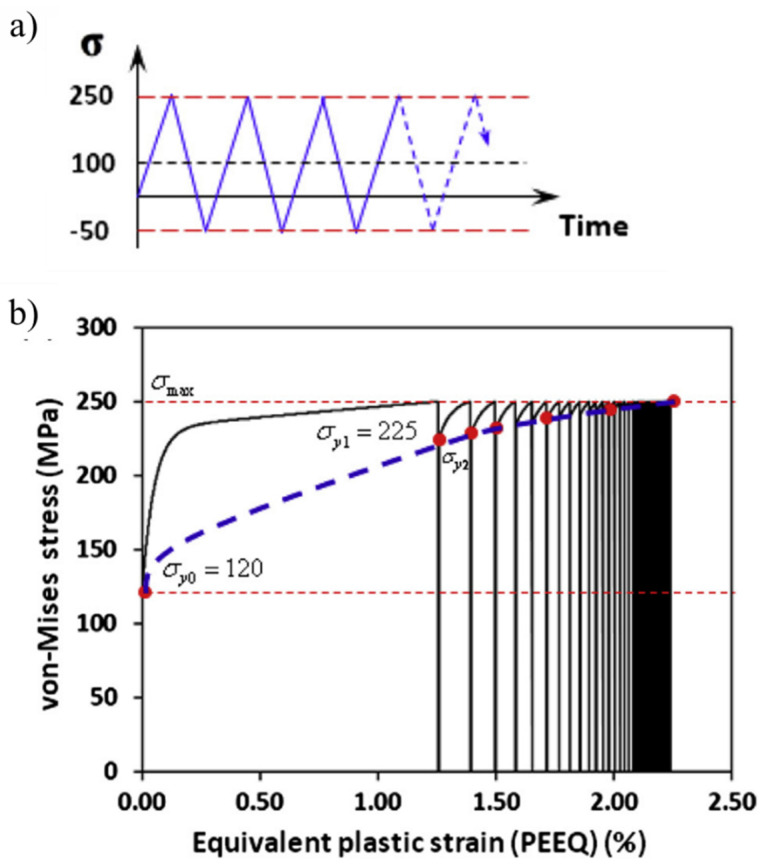
(**a**) Stress-controlled loading for ratcheting simulation employing the NIKH material model; (**b**) cyclic hardening with continuous accumulation of plastic strains [73].

Pandkar et al. [68,73] presented a semi-empirical approach based on combined nonlinear isotropic/kinematic hardening to model the ratcheting response of case hardened bearing steel and to estimate the continuous accumulation of plastic strains in the vicinity of carbides. The cyclic hardening parameters for a case depth of M50-NiL bearing steel were estimated from the subsurface micro-hardness measurement. The NIKH material model employed in this study is based on von Mises yield criterion, with an associative flow rule and an additive decomposition of strain tensor. The generalised associative flow rule for the NIKH material model is given in Equation (1) as [68],

(1) $$f\;\left(S,\;q,{\;\varepsilon }_{\mathrm{pl}}^{-}\right)=\left|\;S-q\;\right|-{\;\sigma }_{y}$$

where ‘S’ is the stress vector, ‘q’ is back-stress tensor,${‘\varepsilon }_{\mathrm{pl}}^{-}’$ is the accumulated plastic strain, and ‘σ_y_’ is the instantaneous yield strength. The depth distribution of the change in hardness of the evolved microstructure of the case-hardened bearing steel was integrated into the NIKH material model. Figure 7 shows the ratcheting response of bearing steel subjected to uniaxial loading in a stress-controlled environment. It can be seen that equivalent or von Mises stresses indicate substantial work hardening, with continuous accumulation of PEEQ. Simulation results showed that the cyclic hardening via ratcheting is promoted at the scale of the carbide microstructure. The hard spheroidised carbide particles act as local stress increasers and are the primary driver for the localised subsurface hardening. A similar approach was extended in a more recent research work [75] in order to simulate the deep zone residual stresses in an evolved bearing steel microstructure. The highly localised subsurface hardness change was incorporated into the Tabor rule [76] to convert the nanoindentation hardness to equivalent flow stresses. The evolved flow stresses were then fed into the newly developed 3D finite model in order to mimic the cyclic hardening response of the evolved bearing steel microstructure.

A continuum damage mechanics (CDM) model was presented by Morris et al. [23], in which microstructural deterioration, material degradation, phase transformation, and resulting residual stress formation were presented. As discussed previously, the development of residual stresses during RCF is associated with the microplasticity and the decay of RA [1,22]. In a 2D rolling fatigue simulation based on CDM [12], Shen et al. presented the effects of RA and compressive residual stresses on the fatigue life of carburised AISI 8620 steel. It was reported that the presence of compressive residual stresses is beneficial for fatigue life by reducing the damage evolution rate. Moreover, the higher amounts of RA resulted in the increased RCF life of carburised steel. In addition, the initial presence of higher RA also leads to prolonged subsurface-initiated spalling life [77]. Morris et al. [23] presented a novel modelling approach to estimate residual stresses as a function of the decay of RA. A CDM model was integrated into a finite element analysis to capture the microstructural deterioration, phase transformation, and residual stress formation. The model was able to predict the residual stress generation under RCF; however, it neglects the plasticity-induced residual stresses during cyclic loadings. 

**Figure 8:**
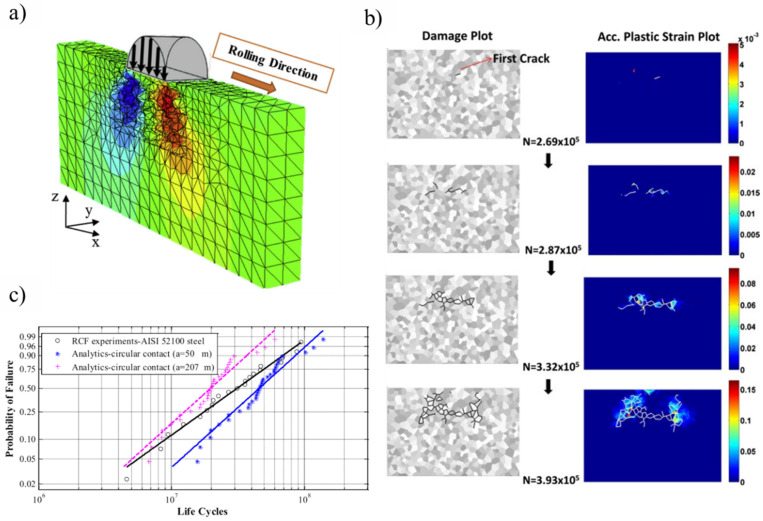
(**a**) A CDM 3D microstructure topology model with Voronoi tessellation; (**b**) crack evolution with the accumulation of plastic strains; (**c**) Weibull probability plots of experimental and analytical results [60,62].

To model the subsurface plasticity and corresponding stress state to initiate the spalling in rolling contact fatigue, a finite element model was integrated with continuum damage mechanics (CDM) [62]. It was reported [13,43,78] that the plastic deformation of the subsurface region during RCF eventually leads to the nucleation of subsurface cracks. A similar approach was utilized by Walvekar [79] in a three-dimensional finite element analysis to report 3D spall formation and the fatigue life scatter of bearing components. A randomly generated Voronoi tessellation technique [80] was implemented to replicate the intergranular paths of the microstructure. A similar meshing technique was implemented in substantial fatigue models [60,62,79,81,82,83,84,85] to imitate the characteristic grain boundary interactions. Figure 8a represents a three-dimensional topology of the circular rolling contact, modelled using the Voronoi tessellation. The evolution of subsurface damage, represented in Figure 8b, indicates subsurface crack initiation and then propagation to form a spall, in conjunction with the continuous accumulation of plastic strains. Figure 8c shows the Weibull probability plot of RCF failure, as acquired from simulation and experimental results. It was testified that the fatigue damage is induced due to the PEEQ around broken intergranular joints leading to crack propagation and eventually, RCF failure.

The cyclic hardening and fatigue life models are based on the constitutive properties of the bearing steel obtained from either the monotonic stress–strain response or the conventional torsional fatigue test and are therefore unable to present a true picture of the multiaxial loading state during RCF [20,29]. Moreover, the cyclic lives of torsional tested steel are seven to nine orders less than the actual life cycle of well-lubricated bearing steel operated under rolling contact conditions [45]. Future work is recommended to incorporate a complex cyclic hardening response with the gradual evolution of bearing steel microstructure. Bridging the gap between the mechanical and metallurgical aspects in modelling RCF would be highly beneficial in estimating residual stresses and the evolved subsurface response.

## 4. Microstructural Modelling

**Figure 9:**
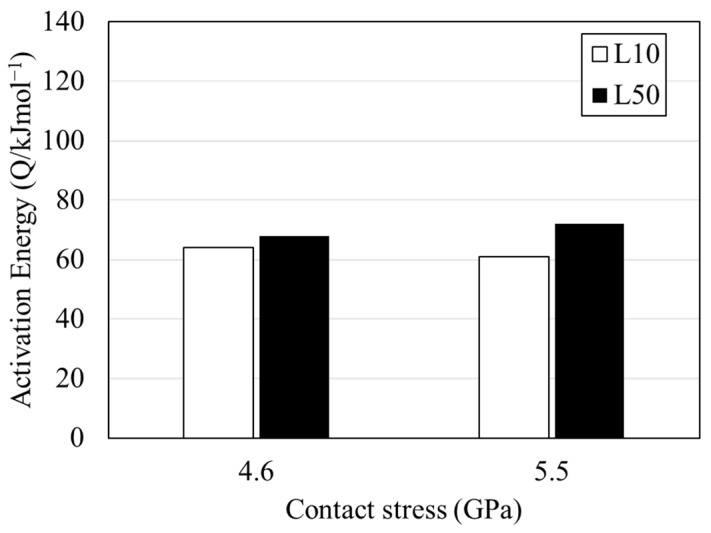
Activation energies of rolling contact fatigue life originated from microstructural alterations at different applied loads (adapted from [88]).

Preliminary investigations [86,87] suggested that microstructural alterations, i.e., DERs and WEBs, are developed due to tempering and localised temperature increase during cyclic loadings. However, no experimental evidence was found to support such an argument. Nevertheless, the threshold stress for such microstructural alterations represents this mechanism to be stress-induced, regardless of bearing running time. The microstructural phase transformation of the martensite (parent material) into ferrite in the affected zones of RCF can be explained by the carbon migration and diffusion theories. Mitamura et al. [88] conducted RCF studies at tempering conditions of bearing steel and reported that the activation energies of the fatigue process (from Arrhenius plots) correspond to those of carbon diffusion in body-centred-cubic (bcc) ferrite, as shown in Figure 9. The experimental and numerical results indicated that the microstructural alterations during RCF are controlled by the diffusion of carbon atoms in the ferrite matrix. Solid state diffusion is classified as vacancy diffusion and interstitial diffusion, where the latter is relevant to structural changes during rolling contact fatigue [89]. During interstitial diffusion, carbon atoms diffuse from one interstitial site to a neighbouring site, without permanently displacing the martensite matrix.

**Figure 10:**
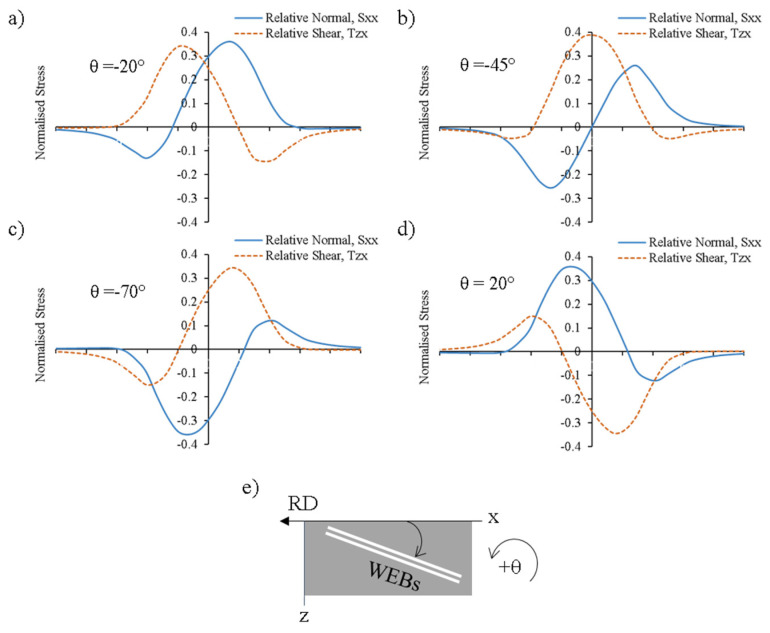
(**a**–**d**) Maximisation of relative normal stress and relative shear stress across various orientations (angle θ) of WEBs; (**e**) represents schematic of WEBs orientation with rolling direction (RD) (adapted from [29]).

The development of WEBs was initially defined as shear bands by K. L. Johnson [90]. It was claimed that the white bands originate at a depth of maximum Hertz shear stress and initiate on planes of maximum shear stress. Polonsky and Keer [15], in a detailed critical analysis, reconstructed the theoretical and analytical models presented by previous researchers [90,91,92] to describe the formation mechanism of white etching bands. It was suggested that the diffusional outflow of carbon from WEBs to form lenticular carbides and ferrite plays a crucial role in WEBs formation. It was reported that the unique orientations of LABs (30°) and HABs (80°) arise from the maximisation of relative normal stress (deviatoric stress) and relative shear stress across the WEBs, instead of from a static state of stress, as suggested previously by Zwirlein et al. [92,93]. A similar theoretical model was presented by Abdullah et al. [94] in recent research work to explain the unique directionalities and formation mechanisms of white bands. Figure 10 represents the distribution of relative normal stress and relative shear stress at various orientations of WEBs. It can be seen that the LABs tend to adopt such orientation, where the relative normal stress maximum is last among all major extrema.

**Figure 11:**
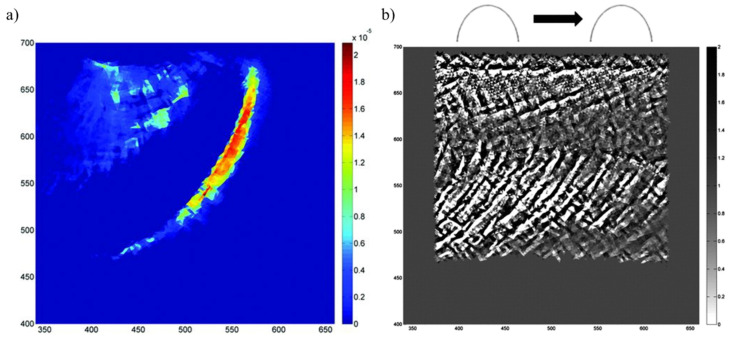
(**a**) Contour plot for dissipated plastic energy per unit volume; (**b**) carbon centration plot as the load moves from left to right in a 2D domain [89].

To develop predictive models for microstructural alterations (DERs/WEBs), a realistic mechanism for the dissolution and redistribution of carbon atoms in a martensite/ferrite matrix is an important aspect. It was suggested by [95] that the development of white bands is a phenomenon of recrystallization, and it involves carbon diffusion resulting in the development of carbon depleted discs (ferrites). These ferrite regions/bands are immune to etching and appear as white contrast bands. Mirza et al. [96] linked thermomechanical and microstructural development with recrystallization and textural evolution during the rolling passes. The carbon from the martensitic matrix becomes trapped at defects (dislocation) or precipitates as cementite bands, creating lenticular carbides. The theory of carbon diffusion during the martensite-ferrite phase transition was supported by previous research work [39,87,97]. Following that, an austenite-ferrite phase transformation model was reported by Pernach et al. [98] which employed a finite difference scheme to solve Fick’s diffusion equation. The model successfully described ferrite volume fraction, carbon segregation and kinetics of phase transformation, but it was based on the pre-existing carbon concentration gradients and hence, was unable to state the true picture of RCF. Zhao et al. [99] presented a sequentially coupled deformation-diffusion analysis to model oxygen diffusion along grain boundaries in a nickel-based superalloy. A crystal plasticity model was used to define the material constitutive behaviour, and the diffusion of oxygen was calculated in the presence of a stress tensor. This stress-assisted diffusion model was further extended by Warhadpande et al. [89] by coupling the stress-assisted diffusion flux with J2 plasticity. It was described that the dissipated plastic strain energy during cyclic plasticity drives the carbon outflow from the parent matrix. Figure 11a shows the distribution of dissipated plastic energy per unit volume as the load moves from left to right in a 2D domain. Correspondingly, the distribution of carbon and unique orientations of WEBs (i.e., LABs and HABs) can be seen in Figure 11b in terms of carbon concentration. It is suggested that during cyclic rolling loadings, carbon migrates in specific orientations in the material domain, corresponding to the directions of WEB. The unique orientation of carbon outflow from the parent matrix represents the formation of 30° and 80° WEBs. The model successfully presents the formation of WEBs orientation; however, it relies on the assumption that material elastic–plastic properties remain homogenous, and that the diffusion coefficient is not affected by temperature or stress variations. 

During RCF loading, the dislocation density can also play a role to drive carbon. As the ferrite phase arises due to the localised plastic deformation of martensite, it contains a significant dislocation density, as reported by Fu et al. [100], and is considered a Cottrell atmosphere [101]. The higher dislocation density can favourably drag carbon atoms and form a strong interaction between nearby dislocation and carbon atoms. During bearing operation, a significant amount of dislocation gliding occurs, which can act as a driving force for carbon migration within the solid solution. Kang et al. [47] presented an analytical model of martensite tempering assisted by dislocation glide during rolling contact fatigue. It was reported that during RCF, the carbon glide according to their atmospheres, enabling carbon to be transported from the matrix to nearby carbides, resulting in carbide thickening within the DER ferrite. The transport of carbon atoms is calculated by considering the interaction between the carbon and the dislocations, binding the energy of the carbon with the carbides. 

**Figure 12:**
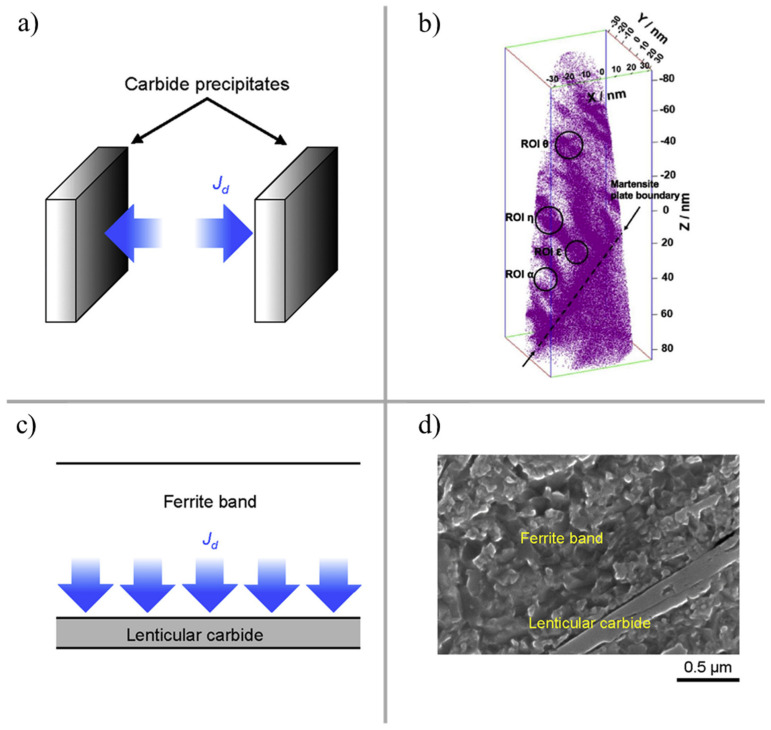
(**a**,**c**) Schematic of carbide thickening within DERs and WEBs; (**b**) experimental evidence for carbon redistribution and precipitation; (**d**) formation of lenticular carbides [103].

Based on the dislocation gliding mechanism, Fu et al. presented strain-induced martensite decay [102] and stress-induced carbide precipitation [48] models to estimate the initiation and growth of DERs and WEBs, respectively. Later on, a unified model [103] for microstructural alterations was presented, suggesting the carbon migration distance as a major aspect of their formation. For dark etching regions, the carbon travels hundreds of nanometres of distance to precipitate with the pre-existing carbides. This thickening of the carbides within the DER zone was confirmed by atomic probe tomography (APT) investigations [102]. For white etching band formation, the carbon migrates from the ferrite matrix and travels several micrometres to develop lenticular carbides [100]. The overall schematic to model microstructural alterations (DER, WEB) is demonstrated in Figure 12. The generalised equations for carbon flux $J_{d}$ equilibria in microstructural alterations are given by [103]

(2) $$J_{d}=\frac{d(r_{p},{\;l}_{\mathrm{LC}})}{\mathrm{dN}}\left(C_{V}^{\mathrm{II}}-C_{V}^{I}\right)\;N{}^{\prime }$$

(3) $$V^{o}C_{V}^{o}={\;V}^{\mathrm{II}}C_{V}^{\mathrm{II}}+(V^{o}-V^{\mathrm{II}}){\;C}_{V}^{I}$$

Equations (2) and (3) can be used to calculate carbon diffusion from zone I to II, where N represents the stress cycles, $r_{p}$ represents the thickness of carbide, $l_{\mathrm{LC}}$ represents the thickening of the lenticular carbides, $V^{o}$ represents the volume of the entire system, and $C_{V}$ denotes carbon concentration per unit volume.

**Figure 13:**
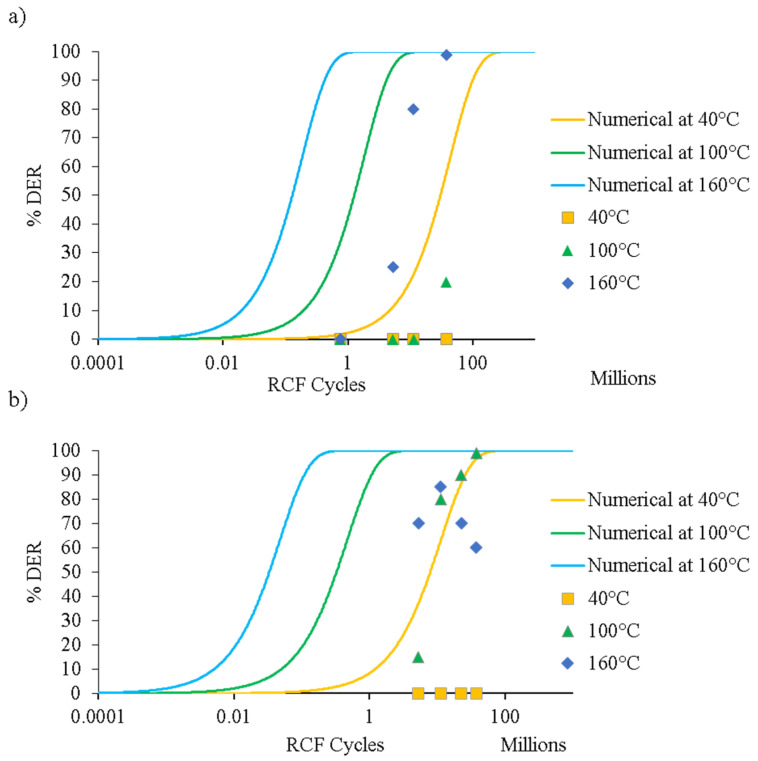
Experimental and numerical prediction of DERs formation at (**a**) 4 GPa and (**b**) 5 GPa contact stress. Adapted from [29].

Abdullah et al. [94,104] presented extensive experimental data on the formation of DERs and WEBs, along with some suggestions to improve existing analytical models. Figure 13 shows the numerical prediction of the formation of DERs with progressive RCF cycles at (a) 4 GPa and (b) 5 GPa contact stress, along with 40 °C, 100 °C, and 160 °C operating temperatures. It was reported that the dislocation gliding model overestimates the formation of microstructural alterations. The main reason for deviation from the experimental data was suggested to be the fact that at extreme loading conditions (near the tempering conditions of bearing steel), the inclusion of carbon diffusivity becomes imperative. It is suggested that the dislocation gliding model is a simplification of carbon migration within the martensitic matrix. According to Fu’s model [102], during DERs formation, carbon migrates from the martensite lattice to the pre-existing tempered state to form θ-Fe_3_C, whereas the initial carbides remain unchanged. APT results have revealed that three forms of carbides coexist in the DER zone, e.g., θ-Fe_3_C, η-Fe_2_C, and ε-Fe_2.4_C [102]. Moreover, the lenticular carbides formed during WEBs have relatively less carbon content, as compared to θ-Fe_3_C [105]. Following extensive experimental investigations, a semi-empirical model for predicting WEBs [106] was presented based on the saturation density of LABs and HABs under a range of contact pressures and stress cycles. Based on the growth pattern of WEBs, it was suggested that the WEBs formation is mainly driven by the diffusion process. In a recent mechanistic study [107], it was reported that both LABs and HABs arise due to recrystallisation from energy build-up in the initial microstructure, which later transforms to the elongated ferrite grains via the grain rotation/coalescence recovery mechanism owing to subsurface plasticity. Mustafa et al. [17] suggested that HABs are formed in the densely formed areas of LABs. However, at elevated temperatures and loads, HABs prior to LABs have also been reported [88,94]. The early formation of HABs suggests that the reversal of the sequence of WEBs (e.g., HABs before LABs) does not depend upon applied load, but rather the temperature-load combination.

It is proposed to couple the existing dislocation gliding models [47,48,102,103] with a more sophisticated elastoplastic model in order to mimic the cyclic hardening response of bearing steel. It has been debated for decades whether the microstructural alterations are governed by the diffusion of carbon or subsurface plastic deformations. It is more likely that the formation mechanism for such structural alterations is affected by carbon diffusion through plastic deformation; however, their individual contributions to the formation mechanism of microstructural alterations is still debatable.

## 5. Overview and Conclusions

The complex heterogenous nature of bearing steel can be realised by chemical composition, non-metallic inclusion, cyclic plasticity, strain hardening/softening, residual stresses evolution and altered microstructure. Modelling the cyclic plasticity and predicting the formation of microstructural alterations become imperative for researchers to understand micromechanical and metallurgical response of bearing steel subjected to RCF. Considerable work has been done so far on the damage mechanics of subsurface-initiated failure to estimate the fatigue life of bearing components; however, limited research has been reported on modelling the evolved microstructure response of the RCF affected zone. 

The continuous accumulation of cyclic plasticity is modelled with the help of elastic-linear kinematic plastic (ELKP), linear/nonlinear kinematic hardening, or combined nonlinear isotropic/kinematic hardening (NIKH) models. The cyclic hardening coefficients for these material models are acquired and calibrated against extensive experimental data. The constitutive response of bearing steel can be characterised by the subsurface hardness measurements of RCF tested samples, which represent the flow stresses of the RCF-affected region. These evolved flow stresses can be incorporated into material models to mimic the cyclic hardening/softening of the bearing steel microstructure.

A more realistic elastic–plastic finite element model considers the nonlinearity of the bearing steel microstructure, along with cyclic plasticity and degradation of the microstructure. It is recommended to integrate the decay of retained austenite in the NIKH material model to evaluate the progression of residual stresses in the later stages of RCF. This will enable the accurate modelling of the deep zone residual stresses in the evolved stress-affected regions with continuously accumulating plastic strains and microstructural phase transformations. Based on the constitutive parameters obtained from RCF testing, a reliable multifaceted fatigue life prediction model can also be developed, incorporating the effects of parent matrix, carbides, inclusions, residual stresses, material evolution/degradation, and the resulting evolved subsurface stress fields.

The analytical and numerical models of microstructural alterations, reported in the literature, are based on carbon diffusion and/or carbide thickening due to the dislocation-assisted carbon migration theory. The carbide thickening models predict the formation of DERs/WEBs in good agreement with the reported experimental data under nominal conditions. However, under extreme conditions, their formation mechanism is greatly influenced by the diffusion process. These analytical models also lack the ability to describe the unique directionalities of the lower angle and higher angle bands. Consequently, a comprehensive understanding is still needed to predict such microstructural alterations. Coupling the three-dimensional elastoplastic models with the dislocation gliding mechanism and integrating the diffusion process is a long-term goal in developing a state-of-the-art prognostic model for microstructural alterations.

## Data Availability

Not applicable.

## References

[1] Kang J.-H., Hosseinkhani B., Rivera-Díaz-del-Castillo P.E. (2012). Rolling contact fatigue in bearings: Multiscale overview. Mater. Sci. Technol..

[2] El Laithy M., Wang L., Harvey T.J., Vierneusel B., Correns M., Blass T.J.T.I. (2019). Further understanding of rolling contact fatigue in rolling element bearings—A review. Tribol. Int..

[3] Voskamp A., Österlund R., Becker P., Vingsbo O. (1980). Gradual changes in residual stress and microstructure during contact fatigue in ball bearings. Met. Technol..

[4] Lundberg G., Palmgren A. (1949). Dynamic Capacity of Rolling Bearings. J. Appl. Mech..

[5] Khurram M., Mufti R.A., Bhutta M.U., Afzal N., Abdullah M.U., Rahman S.U., Rehman S.U., Zahid R., Mahmood K., Ashfaq M. (2020). Roller sliding in engine valve train: Effect of oil film thickness considering lubricant composition. Tribol. Int..

[6] Abdullah M.U., Shah S.R., Bhutta M.U., Mufti R.A., Khurram M., Najeeb M.H., Arshad W., Ogawa K. (2018). Benefits of wonder process craft on engine valve train performance. Proc. Inst. Mech. Eng. Part D J. Automob. Eng..

[7] Klecka M.A., Subhash G., Arakere N.K. (2013). Microstructure–property relationships in M50-NiL and P675 case-hardened bearing steels. Tribol. Trans..

[8] Merwin J., Johnson K. (1963). An analysis of plastic deformation in rolling contact. Proc. Inst. Mech. Eng..

[9] Ruud C. (1982). A review of selected non-destructive methods for residual stress measurement. NDT Int..

[10] Warhadpande A., Sadeghi F., Evans R.D., Kotzalas M.N. (2012). Influence of plasticity-induced residual stresses on rolling contact fatigue. Tribol. Trans..

[11] Khan Z.A., Hadfield M., Tobe S., Wang Y. (2006). Residual stress variations during rolling contact fatigue of refrigerant lubricated silicon nitride bearing elements. Ceram. Int..

[12] Shen Y., Moghadam S.M., Sadeghi F., Paulson K., Trice R.W. (2015). Effect of retained austenite—Compressive residual stresses on rolling contact fatigue life of carburized AISI 8620 steel. Int. J. Fatigue.

[13] Nazir M.H., Khan Z.A., Saeed A. (2018). Experimental analysis and modelling of c-crack propagation in silicon nitride ball bearing element under rolling contact fatigue. Tribol. Int..

[14] Bush J., Grube W., Robinson G. (1961). Microstructural and residual stress changes in hardened steel due to rolling contact. Trans. ASM.

[15] Polonsky I., Keer L. (1995). On white etching band formation in rolling bearings. J. Mech. Phys. Solids.

[16] Šmeļova V., Schwedt A., Wang L., Holweger W., Mayer J. (2017). Electron microscopy investigations of microstructural alterations due to classical Rolling Contact Fatigue (RCF) in martensitic AISI 52100 bearing steel. Int. J. Fatigue.

[17] El Laithy M., Wang L., Harvey T.J., Vierneusel B. (2020). Re-investigation of dark etching regions and white etching bands in SAE 52100 bearing steel due to rolling contact fatigue. Int. J. Fatigue.

[18] Kanetani K., Ushioda K. (2020). Mechanism of White Band (WB) Formation due to Rolling Contact Fatigue in Carburized SAE4320 Steel. Mater. Transations.

[19] Su Y.-S., Li S.-X., Yu F., Lu S.-Y., Wang Y.-G.J.I.J.o.F. (2021). Revealing the shear band origin of white etching area in rolling contact fatigue of bearing steel. Int. J. Fatigue.

[20] Arakere N.K. (2016). Gigacycle rolling contact fatigue of bearing steels: A review. Int. J. Fatigue.

[21] Yin H., Wu Y., Liu D., Zhang P., Zhang G., Fu H.J.M. (2022). Rolling Contact Fatigue-Related Microstructural Alterations in Bearing Steels: A Brief Review. Metals.

[22] Voskamp A. (1985). Material response to rolling contact loading. ASME Trans. J. Tribol..

[23] Morris D., Sadeghi F., Chen Y.-C., Wang C., Wang B. (2018). Effect of Residual Stresses on Microstructural Evolution Due to Rolling Contact Fatigue. J. Tribol..

[24] Cherolis N.E., Benac D.J., Pridemore W.D. (2016). Fatigue in Rotating Equipment: Is it HCF or LCF?. J. Fail. Anal. Prev..

[25] Beswick J.M. (2007). Bearing Steel Technology: Advances and State of the Art in Bearing Steel Quality Assurance: 7th Volume.

[26] Bhadeshia H., Honeycombe R. (2017). Steels: Microstructure and Properties.

[27] Barrow A., Rivera-Díaz-del-Castillo P. (2011). Nanoprecipitation in bearing steels. Acta Mater..

[28] Song W., Choi P.-P., Inden G., Prahl U., Raabe D., Bleck W. (2014). On the spheroidized carbide dissolution and elemental partitioning in high carbon bearing steel 100Cr6. Metall. Mater. Trans. A.

[29] Abdullah M.U. (2022). Finite Element Modelling of Deep Zone Residual Stresses in Rolling Contact Bearing Elements.

[30] Radhakrishnan V., Ramanathan S. (1975). Plastic deformation in rolling contact. Wear.

[31] Lorösch H.-K. (1982). Influence of Load on the Magnitude of the Life Exponent for Rolling Bearings, in Rolling Contact Fatigue Testing of Bearing Steels.

[32] Bhattacharyya A., Pandkar A., Subhash G., Arakere N. (2015). Cyclic constitutive response and effective S–N diagram of M50 NiL case-hardened bearing steel subjected to Rolling Contact Fatigue. J. Tribol..

[33] Turteltaub S., Suiker A. (2005). Transformation-induced plasticity in ferrous alloys. J. Mech. Phys. Solids.

[34] Johnson K. (1989). The strength of surfaces in rolling contact. Proc. Inst. Mech. Eng. Part C Mech. Eng. Sci..

[35] Bhattacharyya A., Subhash G., Arakere N. (2014). Evolution of subsurface plastic zone due to rolling contact fatigue of M-50 NiL case hardened bearing steel. Int. J. Fatigue.

[36] Zaretsky E.V. (2012). Rolling bearing steels—A technical and historical perspective. Mater. Sci. Technol..

[37] Chaboche J.L. (2008). A review of some plasticity and viscoplasticity constitutive theories. Int. J. Plast..

[38] Jhansale H., Topper T. (1971). Engineering Analysis of the Inelastic stress Response of a Structural Metal under Variable Cyclic Strains, in Cyclic Stress-Strain Behavior—Analysis, Experimentation, and Failure Prediction.

[39] Österlund R., Vingsbo O. (1980). Phase changes in fatigued ball bearings. Metall. Mater. Trans. A.

[40] Sadeghi F., Jalalahmadi B., Slack T.S., Raje N., Arakere N.K. (2009). A review of rolling contact fatigue. J. Tribol..

[41] Arakere N., Subhash G. (2012). Work hardening response of M50-NiL case hardened bearing steel during shakedown in rolling contact fatigue. Mater. Sci. Technol..

[42] Jhansale H. (1975). A new parameter for the hysteretic stress-strain behavior of metals. ASME J. Eng. Mater. Technol..

[43] Hahn G., Bhargava V., Rubin C., Chen Q., Kim K. (1987). Analysis of the rolling contact residual stresses and cyclic plastic deformation of SAE 52100 steel ball bearings. J. Tribol..

[44] Swahn H., Becker P., Vingsbo O. (1976). Martensite decay during rolling contact fatigue in ball bearings. Metall. Mater. Trans. A.

[45] Hahn G.T., Bhargava V., Chen Q. (1990). The cyclic stress-strain properties, hysteresis loop shape, and kinematic hardening of two high-strength bearing steels. Metall. Trans. A.

[46] Christ H.J., Sommer C., Mughrabi H., Voskamp A., Beswick J., Hengerer F. (1992). Fatigue behaviour of three variants of the roller bearing steel SAE 52100. Fatigue Fract. Eng. Mater. Struct..

[47] Kang J.-H., Hosseinkhani B., Vegter R.H., Rivera-Díaz-del-Castillo P.E. (2015). Modelling dislocation assisted tempering during rolling contact fatigue in bearing steels. Int. J. Fatigue.

[48] Fu H., Galindo-Nava E., Rivera-Díaz-del-Castillo P. (2017). Modelling and characterisation of stress-induced carbide precipitation in bearing steels under rolling contact fatigue. Acta Mater..

[49] Warhadpande A., Sadeghi F., Evans R.D. (2013). Microstructural alterations in bearing steels under rolling contact fatigue part 1—Historical overview. Tribol. Trans..

[50] Hamilton G. (1963). Plastic flow in rollers loaded above the yield point. Proc. Inst. Mech. Eng..

[51] Bhargava V., Hahn G., Rubin C. (1985). An elastic-plastic finite element model of rolling contact, Part 1: Analysis of single contacts. J. Appl. Mech..

[52] Crook A. (1957). Simulated gear-tooth contacts: Some experiments upon their lubrication and subsurface deformations. Proc. Inst. Mech. Eng..

[53] Nazir M.H., Khan Z.A., Saeed A., Siddaiah A., Menezes P.L. (2018). Synergistic wear-corrosion analysis and modelling of nanocomposite coatings. Tribol. Int..

[54] Howell M., Hahn G., Rubin C., McDowell D. (1995). Finite element analysis of rolling contact for nonlinear kinematic hardening bearing steel. ASME J. Tribol..

[55] Bower A., Johnson K. (1989). The influence of strain hardening on cumulative plastic deformation in rolling and sliding line contact. J. Mech. Phys. Solids.

[56] Jacq C., Nélias D., Lormand G., Girodin D. (2002). Development of a three-dimensional semi-analytical elastic-plastic contact code. J. Trib..

[57] Chaise T., Nélias D. (2011). Contact pressure and residual strain in 3D elasto-plastic rolling contact for a circular or elliptical point contact. J. Tribol..

[58] Chen W.W., Wang Q.J., Wang F., Keer L.M., Cao J. (2008). Three-dimensional repeated elasto-plastic point contacts, rolling, and sliding. J. Appl. Mech..

[59] Walvekar A.A., Sadeghi F. (2017). Rolling contact fatigue of case carburized steels. Int. J. Fatigue.

[60] Golmohammadi Z., Walvekar A., Sadeghi F. (2018). A 3D efficient finite element model to simulate rolling contact fatigue under high loading conditions. Tribol. Int..

[61] Bomidi J.A.R., Sadeghi F. (2013). Three-Dimensional Finite Element Elastic–Plastic Model for Subsurface Initiated Spalling in Rolling Contacts. J. Tribol..

[62] Warhadpande A., Sadeghi F., Kotzalas M.N., Doll G. (2012). Effects of plasticity on subsurface initiated spalling in rolling contact fatigue. Int. J. Fatigue.

[63] Popescu G., Morales-Espejel G.E., Wemekamp B., Gabelli A. (2006). An engineering model for three-dimensional elastic-plastic rolling contact analyses. Tribol. Trans..

[64] Hill R. (1998). The Mathematical Theory of Plasticity.

[65] Prager W. (1956). A new methods of analyzing stresses and strains in work hardening plastic solids. J. Appl. Mech. (ASME).

[66] McDowell D. (1985). A two surface model for transient nonproportional cyclic plasticity, Part 1: Development of appropriate equations. J. Appl. Mech. (ASME).

[67] Mroz Z. (1971). On the Theory of Steady Plastic Cycles in Structures.

[68] Pandkar A.S., Arakere N., Subhash G. (2014). Microstructure-sensitive accumulation of plastic strain due to ratcheting in bearing steels subject to Rolling Contact Fatigue. Int. J. Fatigue.

[69] Chaboche J.-L. (1986). Time-independent constitutive theories for cyclic plasticity. Int. J. Plast..

[70] Kabo E., Ekberg A. (2002). Fatigue initiation in railway wheels—A numerical study of the influence of defects. Wear.

[71] Taraf M., Zahaf E., Oussouaddi O., Zeghloul A. (2010). Numerical analysis for predicting the rolling contact fatigue crack initiation in a railway wheel steel. Tribol. Int..

[72] Portier L., Calloch S., Marquis D., Geyer P. (2000). Ratchetting under tension–torsion loadings: Experiments and modelling. Int. J. Plast..

[73] Pandkar A.S., Arakere N., Subhash G. (2015). Ratcheting-based microstructure-sensitive modeling of the cyclic hardening response of case-hardened bearing steels subject to Rolling Contact Fatigue. Int. J. Fatigue.

[74] Bomidi J.A.R., Weinzapfel N., Slack T., Mobasher Moghaddam S., Sadeghi F., Liebel A., Weber J., Kreis T. (2013). Experimental and Numerical Investigation of Torsion Fatigue of Bearing Steel. J. Tribol..

[75] Abdullah M.U., Khan Z.A., Kruhoeffer W., Blass T. (2020). A 3D Finite Element Model of Rolling Contact Fatigue for Evolved Material Response and Residual Stress Estimation. Tribol. Lett..

[76] Tabor D. (1985). Indentation Hardness and Its Measurement: Some Cautionary Comments, in Microindentation Techniques in Materials Science and Engineering.

[77] Kanetani K., Mikami T., Ushioda K. (2020). Effect of Retained Austenite on Sub-surface Initiated Spalling during Rolling Contact Fatigue in Carburized SAE4320 Steel. ISIJ Int..

[78] Jiang Y., Sehitoglu H. (1996). Modeling of cyclic ratchetting plasticity, part I: Development of constitutive relations. J. Appl. Mech..

[79] Walvekar A.A., Morris D., Golmohammadi Z., Sadeghi F., Correns M. (2018). A Novel Modeling Approach to Simulate Rolling Contact Fatigue and Three-Dimensional Spalls. J. Tribol..

[80] Preparata F.P., Shamos M.I. (2012). Computational Geometry: An Introduction.

[81] Jalalahmadi B., Sadeghi F. (2009). A Voronoi Finite Element Study of Fatigue Life Scatter in Rolling Contacts. J. Tribol..

[82] Raje N., Sadeghi F., Rateick J.R.G., Hoeprich M.R. (2007). A Numerical Model for Life Scatter in Rolling Element Bearings. J. Tribol..

[83] Weinzapfel N., Sadeghi F., Bakolas V. (2010). An approach for modeling material grain structure in investigations of Hertzian subsurface stresses and rolling contact fatigue. J. Tribol..

[84] Weinzapfel N., Sadeghi F., Bakolas V., Liebel A. (2011). A 3D Finite Element Study of Fatigue Life Dispersion in Rolling Line Contacts. J. Tribol..

[85] Shen F., Zhou K. (2019). An elasto-plastic-damage model for initiation and propagation of spalling in rolling bearings. Int. J. Mech. Sci..

[86] Martin J., Borgese S., Eberhardt A. (1966). Microstructural alterations of rolling—Bearing steel undergoing cyclic stressing. ASME J. Basic Eng..

[87] Lund T. (1969). Structural alterations in fatigue-tested ball- bearing steel. Jernkontorets Ann..

[88] Mitamura N., Hidaka H., Takaki S. (2007). Microstructural development in bearing steel during rolling contact fatigue. Mater. Sci. Forum.

[89] Warhadpande A., Sadeghi F., Evans R.D. (2014). Microstructural Alterations in Bearing Steels under Rolling Contact Fatigue: Part 2—Diffusion-Based Modeling Approach. Tribol. Trans..

[90] Johnson K. (1988). Formation of Shear Bands in Ball-Bearing Races.

[91] Bhargava V., Hahn G., Rubin C. (1990). Rolling contact deformation, etching effects, and failure of high-strength bearing steel. Met. Mater Trans A.

[92] Zwirlein O., Schlicht H. (1980). Werkstoffanstrengung bei Wälzbeanspruchung–Einfluß von Reibung und Eigenspannungen. Mater. Und Werkst..

[93] Zwirlein O., Schlicht H. (1982). Rolling Contact Fatigue Mechanisms—Accelerated Testing versus Field Performance, in Rolling Contact Fatigue Testing of Bearing Steels.

[94] Abdullah M.U., Khan Z.A., Kruhoeffer W., Blass T., Vierneusel B. (2021). Development of white etching bands under accelerated rolling contact fatigue. Tribol. Int..

[95] Buchwald J., Heckel R. (1968). An analysis of microstructural changes in 52100 steel bearings during cyclic stressing(Microstructural changes in 52100 steel bearing inner rings during cyclic stressing, obtaining thickening rate data on white-etching regions and lenticular carbides). ASM Trans. Q..

[96] Mirza M., Sellars C., Karhausen K., Evans P. (2001). Multipass rolling of aluminium alloys: Finite element simulations and microstructural evolution. Mater. Sci. Technol..

[97] Lindahl E., Österlund R. (1982). 212 transmission electron microscopy applied to phase transformations in ball bearings. Ultramicroscopy.

[98] Pernach M., Pietrzyk M. (2008). Numerical solution of the diffusion equation with moving boundary applied to modelling of the austenite–ferrite phase transformation. Comput. Mater. Sci..

[99] Zhao L. (2011). Modeling of oxygen diffusion along grain boundaries in a nickel-based superalloy. J. Eng. Mater. Technol..

[100] Fu H., Rivera-Díaz-del-Castillo P. (2019). Evolution of White Etching Bands in 100Cr6 Bearing Steel under Rolling Contact-Fatigue. Metals.

[101] Cottrell A.H., Bilby B. (1949). Dislocation theory of yielding and strain ageing of iron. Proc. Phys. Society. Sect. A.

[102] Fu H., Song W., Galindo-Nava E.I., Rivera-Díaz-del-Castillo P.E. (2017). Strain-induced martensite decay in bearing steels under rolling contact fatigue: Modelling and atomic-scale characterisation. Acta Mater..

[103] Fu H., Rivera-Díaz-del-Castillo P.E. (2018). A unified theory for microstructural alterations in bearing steels under rolling contact fatigue. Acta Mater..

[104] Abdullah M.U., Khan Z.A., Kruhoeffer W. (2020). Evaluation of Dark Etching Regions for Standard Bearing Steel under Accelerated Rolling Contact Fatigue. Tribol. Int..

[105] Fu H. (2017). Microstructural Alterations in Bearing Steels under Rolling Contact Fatigue.

[106] El Laithy M., Wang L., Harvey T.J., Vierneusel B. (2021). Semi-empirical model for predicting LAB and HAB formation in bearing steels. Int. J. Fatigue.

[107] El Laithy M., Wang L., Harvey T.J., Schwedt A., Vierneusel B., Mayer J. (2022). White etching bands formation mechanisms due to rolling contact fatigue. Acta Mater..

